# Echoendoscopic appearance of mediastinal metastasis from papillary renal carcinoma

**DOI:** 10.1111/1759-7714.13704

**Published:** 2020-10-20

**Authors:** Roberto Piro, Roberto Tonelli, Alberto Cavazza, Sofia Taddei, Enrico Clini, Nicola Facciolongo

**Affiliations:** ^1^ Pulmonology Unit, Azienda Unità Sanitaria Locale ‐ IRCCS di Reggio Emilia Reggio Emilia Italy; ^2^ Clinical and Experimental Medicine PhD Program University of Modena Reggio Emilia Reggio Emilia Italy; ^3^ Respiratory Disease Unit, Department of Medical and Surgical Sciences University of Modena Reggio Emilia Reggio Emilia Italy; ^4^ Pathology Unit Azienda Unità Sanitaria Locale ‐ IRCCS di Reggio Emilia Reggio Emilia Italy

**Keywords:** Bronchoscopy, EBUS/TBNA, papillary renal carcinoma, transbronchial needle aspiration

## Abstract

Endobronchial ultrasound‐guided transbronchial needle aspiration (EBUS‐TBNA) is being increasingly used in the diagnostic workup of mediastinal diseases. Here, we report a patient with cystic lesions located in the mediastinum, the sampling of which facilitated the diagnosis of renal neoplasm. This paper confirms the usefulness and safety of EBUS/TBNA on cystic lesions and describes a rare presentation of intrathoracic metastases from carcinoma of the kidney.

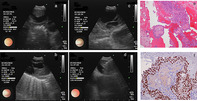

Endobronchial ultrasound bronchoscopy (EBUS) is increasingly used in the diagnostic workup of mediastinal diseases.^1^ When abnormal mediastinal lymph nodes are radiologically detected, the sensitivity of EBUS has been reported to range from 88% to 93%.^1^ In the case reported here, we present the echoendoscopic appearance of mediastinal metastasis from papillary renal carcinoma, stressing the importance of assessment via this ultrasound technique in the diagnostic process.

A 45‐year‐old, Caucasian, former smoker male was admitted to the general ward with a history of considerable weight loss (10 kg over the previous two months) and abdominal pain. His previous medical history did not show anything relevant. When his family history was investigated, he reported that his father had undergone abdominal surgery for a renal tumor at the age of 48, and had died at the age of 78 due to heart failure. Physical examination was not significant. An ultrasound examination followed by a computed tomography (CT) scan of the abdomen was then performed which revealed a left renal mass with a right kidney nodule and multiple abdominal lymphadenopathies. To complete staging, the CT scan was extended to the whole body revealing several mediastinal masses with inhomogeneous appearance (Fig 1). The patient underwent linear probe EBUS that showed the presence of several solid lesions embedded in cysts with a thick wall and anechoic content (Fig 2a‐c); the nodular lesions were located both in the hilar and mediastinal stations (4L, 4R, 7, 10R, 10L and 11R). Ultrasound‐guided transbronchial needle aspiration (EBUS/TBNA) with a 22 G needle in 7 and 11L stations, sampling both the solid and liquid components, which appeared blood tinged macroscopically, and with a total of seven needle passes, were performed (Fig 2d). Cytological examination revealed the presence of several aggregates of moderately atypical epithelial cells, with large eosinophilic cytoplasm, arranged in papillary structures (Fig 2e). Immunohistochemistry analysis showed positivity for PAX8^2^(Fig 2f); TTF1, calretinin and thyroglobulin were negative. Both sampled lymph node stations had the same characteristics. A diagnosis of papillary carcinoma compatible with renal origin was thus made. The patient was treated with sunitinib but died 14 months later.

**Figure 1:**
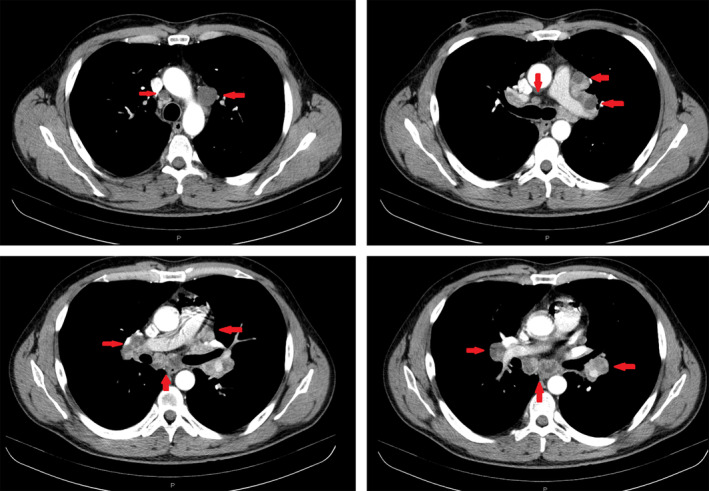
CT images showing the presence of abnormal mediastinal and hilar lymph nodes (red arrows).

Although cystic appearance is not frequent in papillary renal carcinoma,^3^ unilocular cystic masses surrounded by thick‐wall fibrous capsules and filled with hemorrhagic/necrotic contents have been previously reported.^4^ To the best of our knowledge this is the first report to describe ultrasound appearance and diagnosis through transbronchial needle aspiration of mediastinal and hilar metastases of cystic papillary renal carcinoma. Here, we present a rare case of intrathoracic metastases of papillary renal carcinoma in which ecoendoscopic study was essential to highlight the peculiar cystic morphology nature and to perform ultrasound‐guided needle aspiration, which was safe and effective in order to achieve a diagnosis (Fig 2). Given that renal cell carcinoma may metastasize to mediastinal lymph nodes (even without any abdominal lymph node involvement),^5^ it should be considered in the differential diagnosis of mediastinal abnormalities, particularly when a mass is located in the kidneys. In light of strong renal cell carcinoma resistance to chemo‐ and radiotherapy, and its high metastatic index enhanced by hypoxia‐inducible factors (HIFs), the early and precise identification of neoplastic cells, even far from the site of origin, alongside with deep characterization of their biomarkers, may favor efficient therapeutic strategies. This case further confirms the usefulness of EBUS/TBNA in the study of mediastinal and hilar lesions, even in patients with cystic morphology.

**Figure 2:**
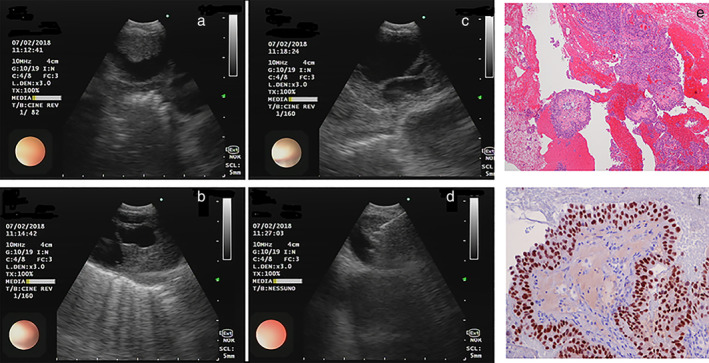
(**a**–**c**) Echoendoscopic images of pathological mediastinal lymph nodes showing the presence of solid structure embedded in cysts with a thick wall and anechoic content. (**d**) Echoendoscopic image of a pathological lymph node showing transbronchial sampling with hyperechoic appearance of the 21 G needle. (**e**) Cytological examination (cell‐block). (**f**) Immunohistochemistry stain showing positivity for PAX8.

## Disclosure

The authors have no conflicts of interest to declare.
